# Association of chronic pain with incidence and progression of cardiometabolic multimorbidity in middle-aged and older populations: a multicohort study

**DOI:** 10.1097/PR9.0000000000001211

**Published:** 2024-12-09

**Authors:** Yating You, Yaguan Zhou, Hui Chen, Angelina Kirilova Kancheva, Rodrigo Martin Carrillo-Larco, Changzheng Yuan, Xiaolin Xu

**Affiliations:** a School of Public Health, The Second Affiliated Hospital, Zhejiang University School of Medicine, Hangzhou, Zhejiang, China; b The Key Laboratory of Intelligent Preventive Medicine of Zhejiang Province, Hangzhou, Zhejiang, China; c School of Health & Wellbeing, University of Glasgow, Glasgow, United Kingdom; d School of Cardiovascular and Metabolic Health, University of Glasgow, Glasgow, United Kingdom; e Emory Global Diabetes Research Centre, Emory University, Atlanta, GA, USA; f Hubert Department of Global Health, Rollins School of Public Health, Emory University, Atlanta, GA, USA; g School of Public Health, Faculty of Medicine, The University of Queensland, Brisbane, Australia

**Keywords:** Chronic pain, Cardiometabolic multimorbidity, Middle-aged and older adults, Multicohort

## Abstract

**Introduction:**

Chronic pain is associated with single cardiometabolic diseases (CMDs). Less is known about the association of chronic pain with the co-occurrence of multiple CMDs, known as cardiometabolic multimorbidity (CMM).

**Objectives:**

This study aims to examine the association between chronic pain and incidence of CMM and if it existed, to what extent chronic pain relates to the progression of specific CMD-related multimorbidity (MM).

**Methods:**

We pooled individual-level data of 59,134 participants from 4 cohort studies across 18 countries between 2010 and 2020. Participants aged 45 years or older, free of CMDs (diabetes, heart diseases, and stroke), and with self-reported chronic pain status at baseline were included. Multinomial logistic regression was performed on the association of chronic pain with incident CMM and the progression of specific CMD-related MM.

**Results:**

One-third (21,204) of participants reported chronic pain at baseline. After 8 to 9 years, 1344 (2.3%) developed CMM. Chronic pain was associated with the onset of each CMD (odds ratio [OR] range 1.12–1.37) and CMM combinations (OR range 1.57–2.09). It is also linked with the increased odds of more CMDs (1, 2, and 3) during the follow-up. For example, OR increased from 1.31 for individuals with one CMD, to 1.57 for those with 2 CMDs, to 2.09 for those with 3 CMDs. Chronic pain was also associated with developing all CMD-related MM (OR range 1.26–1.88). Compared with those with diabetes only, participants with chronic pain were more likely to progress to diabetes and heart diseases, as well as diabetes, heart diseases, and stroke.

**Conclusion:**

Chronic pain is associated with incidence and progression of CMM, whose management should be considered in primary and secondary prevention of CMM among middle-aged and older populations.

## Abstract 2

Supplemental Digital Content is Available in the Text.

Chronic pain management initially needs to be considered as the primary and secondary prevention of cardiometabolic multimorbidity among middle-aged and older population.

## 1. Introduction

Chronic pain, defined as persistent pain beyond 3 months or above, is a common and distressing problem within the ageing population.^1,20^ It affects one-third of people worldwide, 34.1% in 2022.^15^ Chronic pain often accompanies multiple long-term physical conditions, including cardiometabolic diseases (CMDs, eg, diabetes, heart diseases, and stroke), another important global public health concern.^3^ Recently, because of lifestyle changes and increased life expectancy, CMDs have started co-occurring more often, causing cardiometabolic multimorbidity (CMM) to become increasingly prevalent. Cardiometabolic multimorbidity, defined as the coexistence of 2 or more CMDs, has been one of the most replicable multimorbidity profiles worldwide.^2,32^ Cardiometabolic multimorbidity was associated with a higher risk of dementia, disability, and mortality, which might increase burden on limited health resources.^9,30^

A co-twin control study of Spanish Twins found that individuals with chronic low back pain experienced a higher prevalence of myocardial infarction and coronary heart diseases.^7^ This was also supported by a Mendelian randomization analysis, which found a causal relationship between multisite chronic pain and coronary artery disease.^36^ Cross-sectional associations between chronic pain and CMDs have been extensively corroborated, and chronic pain has also been implicated as a risk factor for specific CMDs.^11^ For instance, a prospective study of 45,157 individuals aged 30 to 69 years from the Nord-Trøndelag Health Study found that chronic low back pain was associated with a higher risk of diabetes.^11^ However, rare studies focused on the associations of chronic pain with incident CMM. Only one longitudinal study was found and it showed a significant association between chronic pain and cardiometabolic comorbidity (cardiovascular diseases, with one or more of hypertension, diabetes, and dyslipidemia).^25^ Notwithstanding, results were only limited to Chinese populations. Hence, the associations between chronic pain and CMM within diverse populations warrant further investigation, particularly given current rates of rapid ageing worldwide. Moreover, the pathophysiology of chronic pain is complex where multiple cytokines contribute to the development of CMDs and CMM is potentially harder to treat over time.^8^

Elucidating the association of chronic pain with incident CMM is essential for gaining insights into the importance of chronic pain management and CMM prevention. Herein, we used harmonised data to examine (1) the association between chronic pain and incidence of CMM and (2) if it existed, to what extent chronic pain relates to the progression of specific CMD-related multimorbidity (MM).

## 2. Methods

### 2.1. Study design

In this prospective multicohort study, we identified participants from 4 studies across 18 countries from the Global Aging, Health, and Policy programme^13^: the US Health and Retirement Study (HRS); the China Health and Retirement Longitudinal Study (CHARLS); the English Longitudinal Study on Ageing (ELSA); and the Survey of Health, Ageing and Retirement in Europe (SHARE). These studies belong to the HRS family, featuring matching biennial longitudinal structures and similar survey protocols. They encompass uniform metrics for economic status, lifestyle, and health across nationally representative samples of middle and older adults aged 45 years or older.

### 2.2. Participants

**Figure 1.**
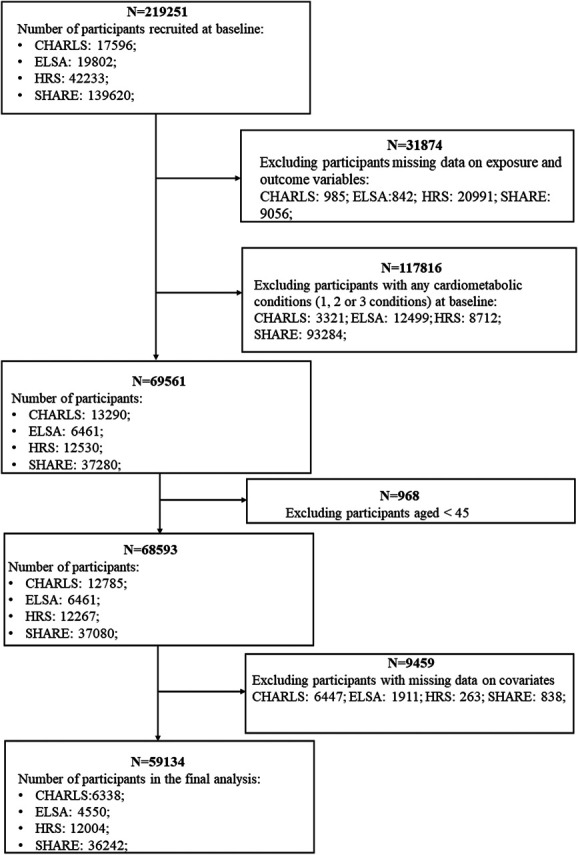
The selection of participants from 4 cohort studies. CHARLS, the China Health and Retirement Longitudinal Study; ELSA, the English Longitudinal Study on Ageing; HRS, the US Health and Retirement Study; SHARE, the Survey of Health, Ageing and Retirement in Europe.

Information on baseline chronic pain status for each study was selected at similar time points (see Table S1, http://links.lww.com/PR9/A269). Participants were included in the analysis if they (1) were 45 years or older, (2) were free from any CMDs at baseline, (3) had data available for baseline chronic pain status, and (4) reported diagnoses of diabetes, heart diseases, and stroke at baseline and during three-wave follow-ups. Participants with missing information on covariates were excluded. All participants gave informed consent. Study selection for all 4 cohort studies is shown in Figure 1.

### 2.3. Definitions

Information on chronic pain was collected through structured interviews at baseline in each cohort. Participants were asked whether they were troubled with frequent pain, and those who responded with a “yes” were recorded as “with chronic pain.”

Cardiometabolic diseases were self-reported through the following question: “Have you ever been diagnosed with these 3 general CMDs by a doctor: (1) diabetes: diabetes or high blood sugar; (2) heart diseases: heart attack, coronary heart disease, angina, congestive heart failure, myocardial infarction, coronary thrombosis, a heart murmur, an abnormal heart rhythm or other heart diseases; and (3) stroke: stroke, transient ischemic attack, and other cerebral vascular diseases?” CMM in this study was defined as coexistence of 2 or 3 CMDs.

To assess CMM incidence, participants were first classified according to number of CMDs (0, 1, 2, and 3) and CMM status (with CMM and without CMM) by last follow-up. Given the different nature of each CMD, participants were further classified into 8 mutually exclusive CMD combinations based on their diagnoses before the last follow-up: (1) no CMDs; (2) diabetes only; (3) heart diseases only; (4) stroke only; (5) diabetes and heart diseases; (6) heart diseases and stroke; (7) diabetes and stroke; and (8) diabetes, heart diseases and stroke. Furthermore, 3 CMD-related MM groups were identified: a diabetes MM group, a heart disease MM group, and a stroke MM group. For instance, using developing diabetes only as the reference group, the diabetes MM group included people developing (1) diabetes and heart diseases; (2) diabetes and stroke; or (3) diabetes, heart diseases and stroke.

Covariates were ascertained at baseline, including age groups (45–54, 55–64, and ≥65 years old), sex, country of origin, body mass index (BMI), marital status, education level, and income level using self-administered questionnaires. Body mass index was quantified as underweight (<18.5 kg/m^2^), normal weight (18.5–24.9 kg/m^2^), overweight (25.0–29.9 kg/m^2^), and obese (≥30 kg/m^2^),^23^ whereas measured BMI from CHARLS was extracted and categorized following the above standard due to the different standards between them. Education level wаs categorized as primary, secondary, and tertiary based on specific criteria in each of the 4 cohorts. Income level was categorized into low, middle, and high according to tertiles.

### 2.4. Statistical analysis

Participant characteristics were described based on baseline chronic pain status. Continuous and categorical variables were presented as mean (standard deviation [SD]) and frequency (percentage), respectively. Group differences were determined using *t* test and χ^2^ test.

Multinomial logistic regression analyses were carried out to examine the association between chronic pain and incidence and progression of CMM. First, the associations of baseline chronic pain with number of CMDs, CMM status, and CMD combinations during follow-up were examined. Second, the association between chronic pain and progression of specific CMD-related MM was investigated, where participants who developed the specific CMD were used as the reference group. For example, in the heart diseases MM group, we analysed the odds of developing (1) heart diseases and diabetes; (2) heart diseases and stroke; and (3) diabetes, heart diseases and stroke, among those with baseline chronic pain as compared with those who developed only heart diseases to explore the impact of chronic pain. We calculated odds ratios (ORs) and 95% confidence intervals (CIs) in crude models (Model 1) and multivariable adjusted models including age and sex (Model 2), and additionally including country, BMI, marital status, education level, and income level (Model 3).

Subgroup analyses were performed to compare the association of chronic pain with CMM among participants stratified by age, sex, and cohort studies as suggested by previous studies.^31^ We further meta-analysed cohort-specific ORs for the association of chronic pain with the incidence of CMM to obtain pooled estimates using random effects models. Between-study heterogeneity was evaluated using the *I*^2^ statistic.

All analyses were conducted using R version 4.2.2 (R Development Core Team, Vienna, Austria) and Stata version 18.0 (Stata Corp Inc., Chicago, IL). A 2-sided *P* < 0.05 was set as statistically significant.

## 3. Results

A total of 59,134 individuals were included in our study. Of them, 12,004 participants (20.3%) were from the United States, 6,338 (10.7%) from China, 4,550 (7.7%) from the United Kingdom, and 36,242 (61.3%) from 15 other European countries (Table S1, http://links.lww.com/PR9/A269). More than one-third of participants reported having chronic pain at baseline in Table 1. Mean age was 63.8 (SD = 9.7) years. There were 34,225 (57.9%) women. Participants who had chronic pain were more likely to be older, female, obese, not married and partnered, have a primary education level, and lower income. Among all participants, 1344 (2.3%) developed CMM, among whom, 1246 (2.1%) and 98 (0.2%) had 2 and 3 CMDs, respectively; 9237 (15.6%) developed one CMD (see Table S2-3, http://links.lww.com/PR9/A269).

**Table 1:** Baseline characteristics of participants according to chronic pain status.

Characteristics	Total (N = 59134)	Chronic pain		*P*
		No (N = 37930)	Yes (N = 21204)	
Age (y, Mean ± SD)	63.8 (9.7)	63.4 (9.6)	64.5 (9.8)	<0.001*
Age groups (y)				<0.001†
45–54	10707 (18.1)	7214 (19.0)	3493 (16.5)	
55–64	23127 (39.1)	15026 (39.6)	8101 (38.2)	
≥65	25300 (42.8)	15690 (41.4)	9610 (45.3)	
Sex				<0.001†
Male	24909 (42.1)	17433 (46.0)	7476 (35.3)	
Female	34225 (57.9)	20497 (54.0)	13728 (64.7)	
BMI				<0.001†
Underweight	1166 (2.0)	705 (1.9)	461 (2.2)	
Normal weight	23604 (39.9)	16033 (42.3)	7571 (35.7)	
Overweight	22756 (38.5)	14804 (39.0)	7952 (37.5)	
Obesity	11608 (19.6)	6388 (16.8)	5220 (24.6)	
Marital status				<0.001†
Married or partnered	44824 (75.8)	29187 (76.9)	15637 (73.7)	
Others	14310 (24.2)	8743 (23.1)	5567 (26.3)	
Education levels				<0.001†
Primary	21910 (37.1)	12793 (33.7)	9117 (43.0)	
Secondary	23805 (40.3)	15474 (40.8)	8331 (39.3)	
Tertiary	13419 (22.7)	9663 (25.5)	3756 (17.7)	
Income levels				<0.001†
Low	19712 (33.3)	11298 (29.8)	8414 (39.7)	
Middle	19704 (33.3)	12483 (32.9)	7221 (34.1)	
High	19718 (33.3)	14149 (37.3)	5569 (26.3)	

* *P* values were from *t* test.

† *P* values were from χ^2^ tests.

BMI, body mass index; SD, standard deviation.

Table 2 shows the association between chronic pain and incident CMM. During 8 to 9 years of follow-up, among participants with chronic pain, 3868 (18.2%) developed one CMD, 588 (2.8%) developed 2 CMDs, and 53 (0.2%) developed 3 CMDs. Chronic pain appeared to be linked with the increased odds of more CMDs (1, 2, and 3) during the follow-up in 3 models. For example, in the fully adjusted model, the ORs were 1.31 (95% CI 1.25–1.38, *P* < 0.001) for one CMD, 1.57 (95% CI 1.40–1.76, *P* < 0.001) for 2 CMDs, and 2.09 (95% CI 1.39–3.15, *P* < 0.001) for 3 CMDs. Compared with participants without chronic pain, those with chronic pain at baseline had 1.51 higher odds of developing CMM (95% CI 1.35–1.69, *P* < 0.001).

**Table 2:** Associations between chronic pain and incident cardiometabolic multimorbidity.

Outcome variable	Cases/No. (%)	Chronic pain, OR (95% CI)		
		Model 1	Model 2	Model 3
Number of CMDs				
0	16695/21204 (78.7)	Ref	Ref	Ref
1	3868/21204 (18.2)	1.37 (1.31–1.44)***	1.39 (1.33–1.45)***	1.31 (1.25–1.38)***
2	588/21204 (2.8)	1.70 (1.52–1.91)***	1.73 (1.54–1.93)***	1.57 (1.40–1.76)***
3	53/21204 (0.2)	2.24 (1.51–3.35)***	2.31 (1.55–3.45)***	2.09 (1.39–3.15)***
†CMM status				
Without CMM	20563/21204 (97.0)	Ref	Ref	Ref
With CMM	641/21204 (3.0)	1.65 (1.48–1.84)***	1.66 (1.49–1.85)***	1.51 (1.35–1.69)***

Model 1: unadjusted. Model 2: adjusted for age and sex. Model 3: adjusted for age, sex, country, body mass index (BMI), marital status, education levels, and income levels.

† CMM was defined as the coexistence of 2 or more types of CMDs (diabetes, heart diseases, or stroke) in this study. ***P < 0.001; **P < 0.01; *, *P* < 0.05.

CI, confidence interval; CMD, cardiometabolic disease; CMM, cardiometabolic multimorbidity; OR, odds ratio.

**Figure 2.**
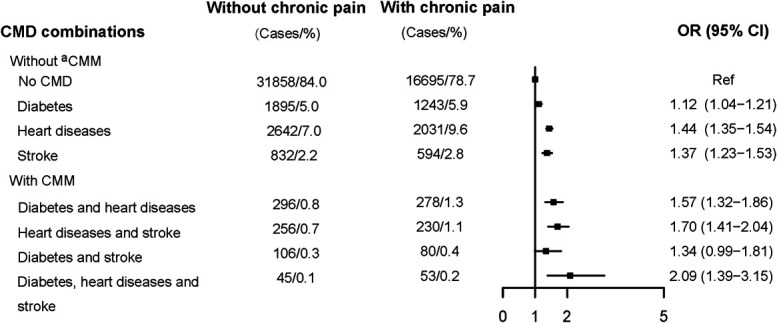
Forest plots for the association between chronic pain and incident cardiometabolic diseases combinations. All models were adjusted for age, sex, country, body mass index (BMI), marital status, education levels, and income levels. Cases/% refers to the frequency and percentage of incident CMD among participants with/without baseline chronic pain. ^a^CMM was defined as the coexistence of 2 or more types of CMDs (diabetes, heart diseases, or stroke) in this study. CMD, cardiometabolic disease; CMM, cardiometabolic multimorbidity; OR, odds ratio; CI, confidence interval.

There were 641 participants (3.0%) with baseline chronic pain developed CMM, of which, diabetes and heart diseases CMM took the largest portion (1.3%) (Fig. 2). We observed significant associations between baseline chronic pain and most CMM combinations, with ORs of 1.57 (95% CI 1.32–1.86, *P* < 0.001) for developing diabetes and heart disease CMM, 1.70 (95% CI 1.41–2.04, *P* < 0.001) for developing heart diseases and stroke CMM, 2.09 (95% CI 1.39–3.15, *P* < 0.001) for developing diabetes, heart diseases, and stroke CMM. Significant associations were also found between chronic pain and single CMDs. For instance, the ORs were 1.12 (95% CI 1.04–1.21, *P* = 0.003) for developing diabetes, 1.37 (95% CI 1.23–1.53, *P* < 0.001) for developing stroke, and 1.44 (95% CI 1.35–1.54, *P* < 0.001) for developing heart diseases.

**Figure 3.**
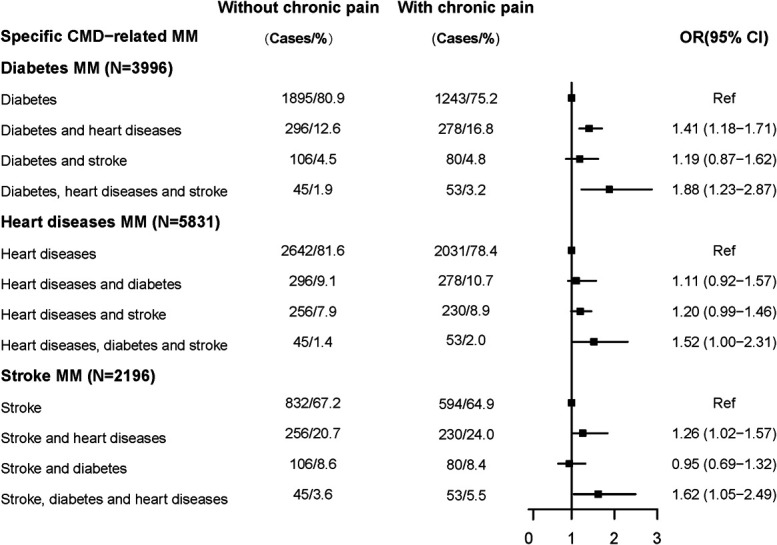
Forest plots for the associations of chronic pain with the progression of specific cardiometabolic diseases–related multimorbidity. The models were adjusted for age, sex, country, body mass index (BMI), marital status, education levels, and income levels. Cases/% refers to the frequency and percentage of incident CMD among participants with/without baseline chronic pain. CI, confidence interval; CMD, cardiometabolic disease; MM, multimorbidity; OR, odds ratio.

Chronic pain was associated with most CMM (Fig. 3). In the diabetes MM group, compared with participants without chronic pain, participants with chronic pain were more likely to progress to diabetes and heart diseases (OR 1.41 95% CI 1.18–1.71, *P* < 0.001), diabetes, heart diseases, and stroke CMM (OR 1.88 95% CI 1.23–2.87, *P* = 0.003) than to progress to diabetes only. In the heart diseases MM group, compared with participants without chronic pain, participants who experienced chronic pain were more likely to develop additional diabetes and stroke (OR 1.52 95% CI 1.00–2.31, *P* = 0.048) than heart diseases only. In the stroke MM group, compared with participants without chronic pain, participants with chronic pain were more likely to progress to additional heart diseases (OR 1.26 95% CI 1.02–1.57, *P* = 0.035) and additional diabetes and heart diseases (OR 1.62 95% CI 1.05–2.49, *P* = 0.028) than to develop stroke alone.

**Figure 4.**
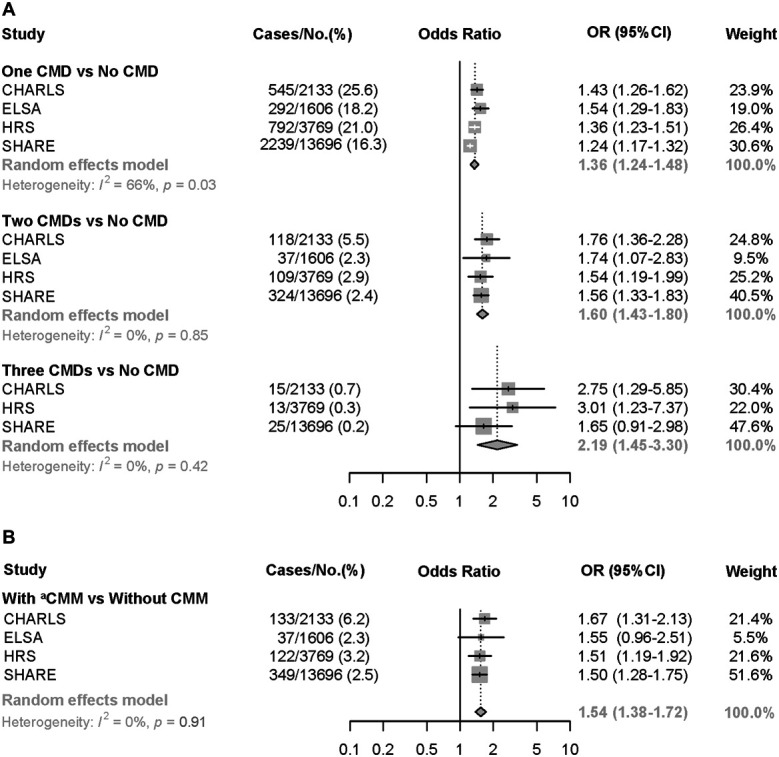
Meta-analysis for the association between baseline chronic pain and (A) number of cardiometabolic diseases and (B) cardiometabolic multimorbidity according to cohort studies. All models were adjusted for age, sex, body mass index (BMI), marital status, education levels, and income levels in CHALRS, ELSA, and HRS cohort, and additionally adjusted for country in SHARE cohort including 15 countries (Austria, Belgium, Czech Republic, Denmark, Estonia, France, Germany, Israel, Italy, Luxembourg, Netherlands, Slovenia, Spain, Sweden and Switzerland). Cases/% refers to the frequency and percentage of incident CMD among participants with/without baseline chronic pain. ^a^CMM was defined as the coexistence of 2 or more types of CMDs (diabetes, heart diseases, or stroke) in this study. CHARLS, the China Health and Retirement Longitudinal Study; CI, confidence interval; CMD, cardiometabolic disease; CMM, cardiometabolic multimorbidity; ELSA, the English Longitudinal Study on Ageing; HRS, the US Health and Retirement Study; OR, odds ratio; SHARE, the Survey of Health, Ageing and Retirement in Europe.

Subgroup analyses stratified by age, sex, and cohort status showed that the odds of developing CMDs among those with chronic pain increased with increasing number of CMDs (Table S4-6, http://links.lww.com/PR9/A269). In addition, the association between chronic pain and CMDs was more pronounced in women than in men, as well as in participants below 65 years compared with those aged 65 years and older. In meta-analysis, pooled ORs (95% CIs) for number of CMDs were 1.36 (1.24–1.48, *I*^*2*^ = 66%) for one CMD, 1.60 (1.43–1.80, *I*^*2*^ = 0%) for 2 CMDs, and 2.19 (1.45–3.30, *I*^*2*^ = 0%) for 3 CMDs, respectively, among those with baseline chronic pain (Fig. 4A). We found significant between-study heterogeneity for the association between baseline chronic pain and developing one CMD. As for dichotomous CMM status, the pooled OR for CMM was 1.54 (1.36–1.71, *I*^*2*^ = 0%) (Fig. 4B).

## 4. Discussion

Our study analysed data from 59,134 middle-aged and older adults from 4 prospective cohort studies across 18 countries with the objective to examine the association of chronic pain with incidence and progression of CMM. Individuals with chronic pain were more likely to develop CMM. The odds of developing CMM increased monotonically with an increased number of CMDs among those with chronic pain. Significant associations were observed between baseline chronic pain and most CMM combinations. Moreover, chronic pain was associated with developing all CMD-related MM. The corresponding associations were more pronounced among women and participants younger than 65 years.

We found significant associations between chronic pain and diabetes, heart disease, and stroke, respectively, consistent with previous studies.^10,14^ A meta-analysis indicated that headache was common at ischemic stroke onset.^10^ A longitudinal study in China demonstrated that chronic pain was independently associated with an increased risk of heart diseases and diabetes.^14^ Moreover, the association between CMDs cannot be ignored. Diabetes is a well-established risk factor for cardiovascular diseases, including heart diseases and stroke.^28^ Conversely, cardiovascular diseases share similar risk factors with diabetes, for example, abdominal obesity, low high-density lipoprotein cholesterol levels, high blood pressure, and elevated levels of fasting glucose.^27^ Hence, CMM might be likely to result from chronic pain and the mutual effects of CMDs. Further research is needed to further clarify the impact of chronic pain and CMDs interactions.

We observed a significant association of chronic pain with CMM incidence, in line with previous studies.^25^ However, this study focused primarily on CMM status rather than specific CMM patterns by the nature of the diseases, providing less targeted evidence. Moreover, its definition of CMM were different from ours. The CMM combinations that we examined were the clinically relatively meaningful CMD combinations and we reported implicated results for major groups of CMM. This could provide more clinical evidence for healthcare professionals. A cross-sectional study conducted in the United Kingdom also found that chronic pain was associated with multiple long-term conditions, eg, diverticular disease, dyspepsia, and mental health,^19^ but this study only covered a few conditions being not representative enough and lacked longitudinal evidence. Our findings extend current evidence on the association of chronic pain with prevalent cardiometabolic health and confirmed chronic pain as an important indicator for the development of CMM.

Our study extends previous research by investigating specific CMD-related MM and provides novel evidence focusing on the association of chronic pain and incidence of 3 CMD-related MM groups. We found that chronic pain potentially impacts CMDs differently, consistent with previous evidence.^16^ This could be due to patients with heart diseases and diabetes potentially perceiving chest pain or discomfort in the chest area differently, as exacerbated by cardiac autonomic neuropathy, which damages autonomic nerve fibres.^17^ In this study, in the diabetes MM group, participants with chronic pain had a higher likelihood of developing diabetes and heart disease CMM than diabetes alone. Moreover, in the stroke MM group, chronic pain was also associated with higher odds of developing the stroke and heart disease CMM than developing stroke only. These findings suggest that chronic pain might have an impact on heart diseases, supported by a meta-analysis showing that greater pain intensity and distribution produces a stronger association with cardiovascular outcomes.^6^

The association between chronic pain and CMDs was more pronounced among women than among men. The differences in their responses to pain could partially explain this finding as responses of women were more variable than those of men, with increased pain sensitivity and many painful diseases commonly reported among women.^24^ The association was also more obvious among participants younger than 65 years than those aged 65 years and older. This might be due to a decrement in pain sensitivity that occurs with ageing^26^; hence, adults younger than 65 years may not be able to tolerate pain and therefore would seek medical attention for their health problems. On the contrary, older people tend to accept chronic pain as part of normal life.^4^ This finding highlighted that attention should be paid to men and old older adults to reduce possible delays in CMD diagnoses.

Mechanisms underpinning the association of chronic pain with incidence and progression of CMDs could possibly be explained by the shared risk factors and pain regulation.^18,22,29,37^ Chronic pain was associated with low levels of physical activity and poorer diet quality.^18,37^ Chronic pain was also associated with anxiety and depression.^22^ These risk factors, unhealthy lifestyle, and mood disorders contributed to the development of CMDs and CMM.^34,35^ Moreover, to defend against pain, the human body releases chemical substances that regulate pain transmission into the extracellular tissue.^29^ Inflammatory biomarkers, such as high-sensitivity C-reactive protein, plasminogen activator inhibitor-1, and other cytokines, have been identified as some predictors of stroke and diabetes.^5,12^

This study is the first to examine the association between chronic pain and clinically meaningful disease combinations, CMM using multicohorts. The strength of this study lies in the large sample size of the multicohort individual data. We focused on a pooled population of middle-aged and older adults from 18 countries across Asia, America, and Europe, providing robust and generalizable evidence on the association of chronic pain with incidence and progression of CMM. Moreover, this study was a prospective cohort study, which corroborates the longitudinal impact of chronic pain on CMM incidence. In addition, multiple classifications of CMM were used, eg, the number of CMDs, CMD combinations, CMM status, and first-identified specific CMM-related MM. Of them, specific CMD-related MM was an innovative method of the CMM category, which provided further insights into assessing CMM focusing not only on the number of disease but also their nature.

Several limitations are noteworthy. First, CMDs were self-reported by participants rather than ascertained by electronic health records, which could have contributed to recall bias. Second, information on the specific sites and severity of chronic pain, as well as severity and subtypes of CMDs, was not provided across 4 cohorts, limiting more detailed analyses on the interaction and targeted relationship between chronic pain and CMDs. Third, none of the 4 cohort studies were from lower-middle and low-income countries. Our findings might thus not be applicable in these countries. Pain is frequently underdiagnosed and undertreated because of poor knowledge about and attitudes toward pain relief, low prioritization of pain management by governments and hospitals, inappropriate legislation, and limited availability of pain treatments in these countries, where similar studies are needed.^21^

Our findings have essential implications for clinical practice and policymaking. First, this study indicated that healthcare professionals should provide timely treatments for chronic pain to prevent the development of CMDs and pay more attention to patients with chronic pain. Second, to achieve the goal of healthy ageing according to WHO's work on ageing between 2015 and 2030, pain management is suggested to be a key primary prevention strategy in preventing CMM because of the high incidence of CMM.^33^ Third, our findings suggested an urgent need to incorporate pain management into future CMM prevention and intervention studies, providing evidence-based guidance and direction. More intervention studies are warranted to optimise pain management programs. Fourth, health commissioners and policymakers should also provide concrete action plans to facilitate the involvement of pain management in CMM prevention as a secondary prevention strategy. This was proposed based on the larger probability of CMD progression to CMM by chronic pain.

## 5. Conclusion

Chronic pain was associated with incidence and progression of CMM. To prevent and slow down CMM progression, health professionals should pay more attention to chronic pain for those at a high risk for CMM. In the context of healthy ageing, chronic pain management should be considered a key priority for primary and secondary prevention of CMM among middle-aged and older populations.

## Disclosures

The authors have no conflict of interest to declare.

## Supplemental digital content

Supplemental digital content associated with this article can be found online at http://links.lww.com/PR9/A269.

## Supplementary Material

SUPPLEMENTARY MATERIAL
