# Supplementary material for: Association of chronic pain with incidence and progression of cardiometabolic multimorbidity in middle-aged and older populations: a multicohort study
Source: Pain Rep. 2024 Dec 9;10(1):e1211. doi: 10.1097/PR9.0000000000001211 (PMC11630955; doi:10.1097/PR9.0000000000001211)
Supplement: SUPPLEMENTARY MATERIAL [file painreports-10-e1211-s001.pdf]

Supplementary information

Table S1. Characteristics of participants in each study by country

Table S2. Characteristics of participants according to number of cardiometabolic diseases and cardiometabolic multimorbidity status

Table S3. Characteristics of participants according to number of cardiometabolic diseases combinations

Table S4. Subgroup analyses for the association between baseline chronic pain and the number of cardiometabolic diseases and cardiometabolic multimorbidity status by age and sex

Table S5. Subgroup analyses for the association between chronic pain and incident cardiometabolic multimorbidity combinations by age and sex

Table S6. Subgroup analyses for the association between baseline chronic pain and incident cardiometabolic multimorbidity by study

Table S1. Characteristics of participants in each study by country

Country	Study	Baseline year for chronic pain	Follow-up waves	Number of participants (Total/female)	Baseline age (years) (mean [SD])
Austria	SHARE	2012-2013	3	2387/1453	65.0(9.2)
Belgium	SHARE	2012-2013	3	3265/1875	63.3(9.6)
China	CHARLS	2011-2012	3	6338/3233	60.4(9.4)
Czech Republic	SHARE	2012-2013	3	2907/1817	64.2(8.4)
Denmark	SHARE	2012-2013	3	2675/1500	63.0(9.4)
Estonia	SHARE	2012-2013	3	2869/1761	65.2(9.1)
France	SHARE	2012-2013	3	2453/1496	65.0(9.7)
Germany	SHARE	2012-2013	3	3235/1821	62.2(9.3)
Israel	SHARE	2012-2013	3	1099/671	64.1(8.8)
Italy	SHARE	2012-2013	3	2851/1656	64.4(9.4)
Luxembourg	SHARE	2013-2014	3	937/520	62.9(9.3)
Netherlands	SHARE	2012-2013	3	1320/783	63.5(7.9)
Slovenia	SHARE	2012-2013	3	1663/1007	64.1(9.3)
Spain	SHARE	2012-2013	3	3725/2102	65.3(10.2)
Sweden	SHARE	2012-2013	3	2732/1536	66.5(8.8)
Switzerland	SHARE	2012-2013	3	2124/1228	64.9(9.3)
United Kingdom	ELSA	2010-2011	3	4550/2515	65.5(8.4)
United States	HRS	2010-2011	3	12004/7251	63.4(10.7)

Notes: Abbreviations: CHARLS, the China Health and Retirement Longitudinal Study; ELSA, the English Longitudinal Study on Ageing; HRS, the US Health and Retirement Study; SHARE, the Survey of Health, Ageing and Retirement in Europe.

Table S2. Characteristics of participants according to number of cardiometabolic diseases and cardiometabolic multimorbidity status

Characteristics	Total (N=59134)	Number of CMDs				P value	CMM		P value
		0 (N=48553)	1 (N=9237)	2 (N=1246)	3 (N=98)		Without [a]CMM (N=57790)	With CMM (N=1344)	
**Age (yrs)**						<0.001			<0.001
45-54	10707 (18.1)	9390 (19.3)	1186 (12.8)	123 (9.9)	8 (8.2)		10576 (18.3)	131 (9.7)	
55-64	23127 (39.1)	19630 (40.4)	3080 (33.3)	389 (31.2)	28 (28.6)		22710 (39.3)	417 (31.0)	
65 and above	25300 (42.8)	19533 (40.2)	4971 (53.8)	734 (58.9)	62 (63.3)		24504 (42.4)	796 (59.2)	
**Sex**						<0.001			<0.001
Male	24909 (42.1)	19968 (41.1)	4295 (46.5)	596 (47.8)	50 (51.0)		24263 (42.0)	646 (48.1)	
Female	34225 (57.9)	28585 (58.9)	4942 (53.5)	650 (52.2)	48 (49.0)		33527 (58.0)	698 (51.9)	
**BMI**						<0.001			<0.001
Underweight	1166 (2.0)	986 (2.0)	161 (1.7)	19 (1.5)	0 (0.0)		1147 (2.0)	19 (1.4)	
Normal weight	23604 (39.9)	20140 (41.5)	3062 (33.1)	373 (29.9)	29 (29.6)		23202 (40.1)	402 (29.9)	
Overweight	22756 (38.5)	18635 (38.4)	3599 (39.0)	484 (38.8)	38 (38.8)		22234 (38.5)	522 (38.8)	
Obesity	11608 (19.6)	8792 (18.1)	2415 (26.1)	370 (29.7)	31 (31.6)		11207 (19.4)	401 (29.8)	
**Marital status**						<0.001			0.001
Married or partnered	44824 (75.8)	37152 (76.5)	6706 (72.6)	905 (72.6)	61 (62.2)		43858 (75.9)	966 (71.9)	
Others	14310 (24.2)	11401 (23.5)	2531 (27.4)	341 (27.4)	37 (37.8)		13932 (24.1)	378 (28.1)	
**Education levels**						<0.001			<0.001
Primary	21910 (37.1)	17214 (35.5)	3995 (43.2)	642 (51.5)	59 (60.2)		21209 (36.7)	701 (52.2)	
Secondary	23805 (40.3)	19777 (40.7)	3563 (38.6)	436 (35.0)	29 (29.6)		23340 (40.4)	465 (34.6)	
Tertiary	13419 (22.7)	11562 (23.8)	1679 (18.2)	168 (13.5)	10 (10.2)		13241 (22.9)	178 (13.2)	
**Income levels**						<0.001			<0.001
Low	19712 (33.3)	15471 (31.9)	3645 (39.5)	541 (43.4)	55 (56.1)		19116 (33.1)	596 (44.3)	
Middle	19704 (33.3)	16185 (33.3)	3077 (33.3)	419 (33.6)	23 (23.5)		19262 (33.3)	442 (32.9)	
High	19718 (33.3)	16897 (34.8)	2515 (27.2)	286 (23.0)	20 (20.4)		19412 (33.6)	306 (22.8)	
**Chronic pain**						<0.001			<0.001
Without pain	37930 (64.1)	31858 (65.6)	5369 (58.1)	658 (52.8)	45 (45.9)		37227 (64.4)	703 (52.3)	
With pain	21204 (35.9)	16695 (34.4)	3868 (41.9)	588 (47.2)	53 (54.1)		20563 (35.6)	641 (47.7)	

Notes: Data are n (%), unless otherwise indicated. P values were from chi-squared tests. Abbreviations: BMI, body mass index; CMD, cardiometabolic disease; CMM, cardiometabolic multimorbidity. [a] CMM was defined as the coexistence of two or more types of CMDs (diabetes, heart diseases or stroke) in the present study.

Table S3. Characteristics of participants according to number of cardiometabolic diseases combinations

Characteristics	Total (N=59134)	CMD combinations								P value
						With [a] CMM				
		No CMDs (N=48553)	Diabetes (N=3138)	Heart diseases (N=4673)	Stroke (N=1426)	Diabetes and heart problems (N=574)	Heart diseases and stroke (N=486)	Diabetes and stroke (N=186)	Diabetes, heart diseases and stroke (N=98)	
**Age (yrs)**										<0.001
45-54	10707 (18.1)	9390 (19.3)	577 (18.4)	462 (9.9)	147 (10.3)	70 (12.2)	30 (6.2)	23 (12.4)	8 (8.2)	
55-64	23127 (39.1)	19630 (40.4)	1268 (40.4)	1424 (30.5)	388 (27.2)	206 (35.9)	114 (23.5)	69 (37.1)	28 (28.6)	
65 and above	25300 (42.8)	19533 (40.2)	1293 (41.2)	2787 (59.6)	891 (62.5)	298 (51.9)	342 (70.4)	94 (50.5)	62 (63.3)	
**Sex**										<0.001
Male	24909 (42.1)	19968 (41.1)	1395 (44.5)	2211 (47.3)	689 (48.3)	262 (45.6)	239 (49.2)	95 (51.1)	50 (51.0)	
Female	34225 (57.9)	28585 (58.9)	1743 (55.5)	2462 (52.7)	737 (51.7)	312 (54.4)	247 (50.8)	91 (48.9)	48 (49.0)	
**BMI**										<0.001
Underweight	1166 (2.0)	986 (2.0)	29 (0.9)	98 (2.1)	34 (2.4)	9 (1.6)	7 (1.4)	3 (1.6)	0 (0.0)	
Normal weight	23604 (39.9)	20140 (41.5)	674 (21.5)	1745 (37.3)	643 (45.1)	127 (22.1)	199 (40.9)	47 (25.3)	29 (29.6)	
Overweight	22756 (38.5)	18635 (38.4)	1220 (38.9)	1859 (39.8)	520 (36.5)	240 (41.8)	174 (35.8)	70 (37.6)	38 (38.8)	
Obesity	11608 (19.6)	8792 (18.1)	1215 (38.7)	971 (20.8)	229 (16.1)	198 (34.5)	106 (21.8)	66 (35.5)	31 (31.6)	
**Marital status**										<0.001
Married or partnered	44824 (75.8)	37152 (76.5)	2307 (73.5)	3390 (72.5)	1009 (70.8)	432 (75.3)	338 (69.5)	135 (72.6)	61 (62.2)	
Others	14310 (24.2)	11401 (23.5)	831 (26.5)	1283 (27.5)	417 (29.2)	142 (24.7)	148 (30.5)	51 (27.4)	37 (37.8)	
**Education levels**										<0.001
Primary	21910 (37.1)	17214 (35.5)	1340 (42.7)	1971 (42.2)	684 (48.0)	327 (57)	216 (44.4)	99 (53.2)	59 (60.2)	
Secondary	23805 (40.3)	19777 (40.7)	1289 (41.1)	1773 (37.9)	501 (35.1)	194 (33.8)	184 (37.9)	58 (31.2)	29 (29.6)	
Tertiary	13419 (22.7)	11562 (23.8)	509 (16.2)	929 (19.9)	241 (16.9)	53 (9.2)	86 (17.7)	29 (15.6)	10 (10.2)	
**Income levels**										<0.001
Low	19712 (33.3)	15471 (31.9)	1260 (40.2)	1800 (38.5)	585 (41)	266 (46.3)	202 (41.6)	73 (39.2)	55 (56.1)	
Middle	19704 (33.3)	16185 (33.3)	1022 (32.6)	1594 (34.1)	461 (32.3)	184 (32.1)	161 (33.1)	74 (39.8)	23 (23.5)	
High	19718 (33.3)	16897 (34.8)	856 (27.3)	1279 (27.4)	380 (26.6)	124 (21.6)	123 (25.3)	39 (21.0)	20 (20.4)	
**Chronic pain**										<0.001
Without pain	37930 (64.1)	31858 (65.6)	1895 (60.4)	2642 (56.5)	832 (58.3)	296 (51.6)	256 (52.7)	106 (57.0)	45 (45.9)	
With pain	21204 (35.9)	16695 (34.4)	1243 (39.6)	2031 (43.5)	594 (41.7)	278 (48.4)	230 (47.3)	80 (43.0)	53 (54.1)	

Notes: Data are n (%), unless otherwise indicated. P values were from chi-squared tests. Abbreviations: BMI, body mass index; CMD, cardiometabolic disease; CMM, cardiometabolic multimorbidity. [a] CMM was defined as the coexistence of two or more types of CMDs (diabetes, heart diseases or stroke) in the present study.

Table S4. Subgroup analyses for the association between baseline chronic pain and the number of cardiometabolic diseases and cardiometabolic multimorbidity status by age and sex

		Number of CMDs				CMM Status	
**Covariates**	**Cases/No. (%)**	**0**	**1**	**2**	**3**	**Without [a] CMM**	**With CMM**
**Age**							
Below 65	11594/33834 (34.3)	Ref	1.41 (1.31, 1.50)	1.66 (1.39, 1.99)	2.21 (1.13, 4.32)	Ref	1.59 (1.34, 1.90)
65 and above	9610/25300 (38.0)	Ref	1.22 (1.14, 1.30)	1.50 (1.29, 1.75)	1.94 (1.16, 3.25)	Ref	1.46 (1.27, 1.69)
**Sex**							
Male	7476/24909 (30.0)	Ref	1.25 (1.16, 1.34)	1.42 (1.20, 1.68)	1.50 (0.84, 2.67)	Ref	1.36 (1.15, 1.60)
Female	13728/34225 (40.1)	Ref	1.36 (1.28, 1.45)	1.73 (1.47, 2.03)	3.02 (1.62, 5.62)	Ref	1.68 (1.44, 1.96)

Notes: Data are OR (95%CI), unless otherwise indicated. All models were adjusted for age, sex, country, body mass index (BMI), marital status, education levels, and income levels. Abbreviations: BMI, body mass index; CMD, cardiometabolic disease; CMM, cardiometabolic multimorbidity; OR, odds ratio; CI, confidence interval. [a] CMM was defined as the coexistence of two or more types of CMDs (diabetes, heart diseases or stroke) in the present study.

Table S5. Subgroup analyses for the association between chronic pain and incident cardiometabolic multimorbidity combinations by age and sex

		CMD combinations							
						With [a] CMM			
**Covariates**	**Cases/No. (%)**	**No CMD**	**Diabetes**	**Heart diseases**	**Stroke**	**Diabetes and heart diseases**	**Heart diseases and stroke**	**Diabetes and stroke**	**Diabetes, heart diseases and stroke**
**Age**									
Below 65	11594/33834 (34.3)	Ref	1.23(1.12, 1.36)	1.61(1.46, 1.77)	1.38(1.16, 1.66)	1.75(1.37, 2.24)	1.87(1.33, 2.62)	1.18(0.76, 1.82)	2.21(1.13, 4.34)
65 and above	9610/25300 (38.0)	Ref	0.96(0.85, 1.08)	1.33(1.22, 1.45)	1.34(1.16, 1.54)	1.38(1.08, 1.75)	1.60(1.28, 2.00)	1.50(0.98, 2.29)	1.91(1.13, 3.21)
**Sex**									
Male	7476/24909 (30.0)	Ref	1.08(0.95, 1.21)	1.38(1.25, 1.52)	1.31(1.11, 1.54)	1.35(1.04, 1.75)	1.65(1.26, 2.15)	1.23(0.80, 1.89)	1.52(0.85, 2.70)
Female	13728/34225 (40.1)	Ref	1.17(1.05, 1.29)	1.51(1.39, 1.65)	1.43(1.23, 1.67)	1.80(1.43, 2.28)	1.77(1.37, 2.29)	1.48(0.96, 2.26)	3.00(1.61, 5.62)

Notes: Data are OR (95%CI), unless otherwise indicated. All models were adjusted for age, sex, country, body mass index (BMI), marital status, education levels, and income levels. Abbreviations: BMI, body mass index; CMD, cardiometabolic disease; CMM, cardiometabolic multimorbidity; OR, odds ratio; CI, confidence interval. [a] CMM was defined as the coexistence of two or more types of CMDs (diabetes, heart diseases or stroke) in the present study.

Table S6. Subgroup analyses for the association between baseline chronic pain and incident cardiometabolic multimorbidity by study

	CHARLS (N=6338, from 2011 to 2019)		ELSA (N=4550, from 2010 to 2018)		HRS (N=12004, from 2010 to 2018)		SHARE (N=36242, from 2012 to 2020)	
	Cases/No. (%)	OR (95%CI)	Cases/No. (%)	OR (95%CI)	Cases/No. (%)	OR (95%CI)	Cases/No. (%)	OR (95%CI)
**Number of CMDs**								
**0**	1455/2133 (68.2)	Ref	1277/1606 (79.5)	Ref	2855/3769 (75.7)	Ref	11108/13696 (81.1)	Ref
**1**	545/2133 (25.6)	1.43 (1.26, 1.62)	292/1606 (18.2)	1.54 (1.29, 1.83)	792/3769 (21.0)	1.36 (1.23, 1.51)	2239/13696 (16.3)	1.24 (1.17, 1.32)
**2**	118/2133 (5.5)	1.76 (1.36, 2.28)	37/1606 (2.3)	1.74 (1.07, 2.83)	109/3769 (2.9)	1.54 (1.19, 1.99)	324/13696 (2.4)	1.56 (1.33, 1.83)
**3**	15/2133 (0.7)	2.75 (1.29, 5.85)	-	-	13/3769 (0.3)	3.01 (1.23, 7.37)	25/13696 (0.2)	1.65 (0.91, 2.98)
**[a]CMM Status**								
Without CMM	2000/2133 (93.8)	Ref	1569/1606 (97.7)	Ref	3647/3769 (96.8)	Ref	13347/13696 (97.5)	Ref
With CMM	133/2133 (6.2)	1.67 (1.31, 2.13)	37/1606 (2.3)	1.55 (0.96, 2.51)	122/3769 (3.2)	1.51 (1.19, 1.92)	349/13696 (2.5)	1.50 (1.28, 1.75)

Notes: All models were adjusted for age, sex, body mass index (BMI), marital status, education levels, and income levels in CHALRS, ELSA and HRS cohort, and additionally adjusted for country in SHARE cohort including 15 countries (Austria, Belgium, Czech Republic, Denmark, Estonia, France, Germany, Israel, Italy, Luxembourg, Netherlands, Slovenia, Spain, Sweden, and Switzerland). [a] CMM was defined as the coexistence of two or more types of CMDs (diabetes, heart diseases or stroke) in the present study. Abbreviations: CMD, cardiometabolic disease; CMM, cardiometabolic multimorbidity; OR, odds ratio; CI, confidence interval. CHARLS, the China Health and Retirement Longitudinal Study; ELSA, the English Longitudinal Study on Ageing; HRS, the US Health and Retirement Study; SHARE, the Survey of Health, Ageing and Retirement in Europe.
